# Shape Memory Behaviour of PMMA-Coated NiTi Alloy under Thermal Cycle

**DOI:** 10.3390/polym14142932

**Published:** 2022-07-20

**Authors:** Sneha Samal, Olga Kosjakova, David Vokoun, Ivo Stachiv

**Affiliations:** FZU—Institute of Physics of Czech Academy of Science, Prague 8, Na Slovance 1999/2, 18221 Prague, Czech Republic; kosjakova@fzu.cz (O.K.); vokoun@fzu.cz (D.V.); stachiv@fzu.cz (I.S.)

**Keywords:** curvature, radius, shape recovery, NiTi, PMMA, thermal cycles

## Abstract

Both poly(methyl methacrylate) (PMMA) and NiTi possess shape memory and biocompatibility behavior. The macroscale properties of PMMA–NiTi composites depend immensely on the quality of the interaction between two components. NiTi shape memory alloy (SMA) and superelastic (SE) sheets were spin coated on one side with PMMA. The composite was prepared by the spin coating method with an alloy-to-polymer-thickness ratio of 1:3. The bending stiffness and radius of curvature were calculated by using numerical and experimental methods during thermal cycles. The experimental radius curvatures in actuation have good agreement with the model. The change in shape results from the difference in coefficients of thermal expansion between PMMA and NiTi. Actuation temperatures were between 0 and 100 °C for the SMA–PMMA composite with a change in curvature from 10 to 120 mm with fixed Young’s modulus of PMMA at 3 GPa, and a change in Young’s modulus of NiTi from 30 to 70 GPa. PMMA–NiTi composites are useful as actuators and sensor elements.

## 1. Introduction

**Figure 1:**
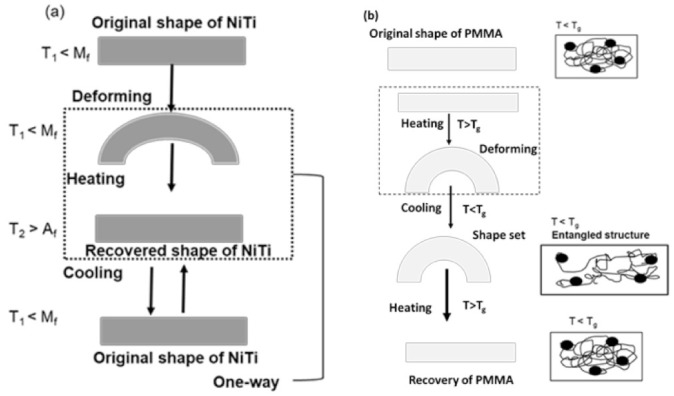
(**a**) One-way shape memory effect of NiTi alloy; (**b**) Shape change and recovery of PMMA.

Thermo-responsive shape memory polymers are those which have the capability of changing their shape from temporary phase to a permanent shape upon application of an external thermal stimulus. Advantages such as highly tunable properties, lower density, easier processing, and lower cost provide great potential for applications in biomedicine, intelligent textiles, and aerospace engineering. PMMA is the scientific name for the synthetic polymer commonly known as acrylic glass, which is transparent, light weight and carries the property of shape memory behavior. Shape memory materials can recover their original shape after being severely and quasi-plastically distorted when the right stimulus is applied [1]. This phenomenon is known as the shape memory effect (SME), and it has been found in several material systems, including alloys, polymers and ceramics [2,3,4]. The procedure to fix the temporary distorted shape is known as programming within the shape memory materials. Polymers with SME properties are considered shape memory polymers (SMP). Thermal stimulus is applied to induce the shape memory effect in SMP materials. Thin films composite of NiTi alloys have attracted a lot of intention in biomedical industries due to their unique properties of shape memory effect and superelasticity with deformation capability, damping capacity, and biocompatibility [5,6,7,8,9]. Shape memory effects can be classified into one-way shape memory effect and two-way shape memory effect (TWSME). In low temperature regions, after an SMA is loaded and unloaded, some residual strain remains. However, if the temperature is raised to a specific temperature, the residual strain vanishes and the SMA returns to its original shape. This phenomenon is known as the one-way shape memory effect. The two-way shape memory effect (TWSME) represents a reversible spontaneous shape change during the cooling and heating process. This is the consequence of reversible phase transformations observed without application of any external force. The shape memory effect of NiTi alloy and PMMA are shown in Figure 1a,b. In shape memory alloys at twinned martensitic finish temperature (T_1_ < M_f_), which allows phase transformation from martensite to detwinned martensitic form for deformed shape as temporary form. Upon heating, reverse phase transformation occurs from detwinned martensite to austenite, which allows the restoration of the original shape (T_2_ > A_f_) and also allows cooling at room temperature to martensite phase (T_1_ < M_f_). This phenomenon is described for NiTi alloy as one-way shape memory effect in Figure 1a.

PMMA has revealed shape memory properties to some extent. PMMA foil could be able to bend on external load and return on release of load. The polymer heated above the glass transition temperature softens and can be deformed to a temporary shape and then return to its original shape upon cooling. Like shape memory alloys, shape memory polymers (SMPs) are attracting attention for their use in wearable displays and biomedical applications, due to their good biocompatibility and excellent moldability [10,11,12]. PMMA is considered one of the shape memories alloys that has the advantage of being lightweight with excellent shape recovery due to their low density [13]. The heating response of polymer above the glass transition temperature (T_g_) makes deformation easier, since the polymer converts into a rubbery state followed by unloading to remain to constrain the distorted shape is largely maintained. Since NiTi and PMMA share several properties such as shape memory behavior, deformability, and bio-compatibility, they are good candidates for a thin-film composite. Several attempts have been made toward the application of integrated NiTi polymer composite in the fields of robotics, micro fluids, and pneumatic application. In biomedical industries, NiTi is used in various sectors of the human body. It is widely used in electronic skin, neurogrid and robotic hand [14,15,16,17]. Additionally, NiTi is applied in the fields of sensors and actuators, e.g., in E-capsules, micro-needles, and bladder actuators [9,18,19]. Shape memory composites are transformed by applying an external force at a high temperature, and the shape is temporarily fixed when it cooled. Later the composite returns to its original, permanent shape at a temperature above the glass transition temperature. The shape change results from the difference in coefficients of thermal expansion and from the change of Young’s modulus and of the coefficient of thermal expansion in the NiTi SMA [20].

The limited rigidity and processing conditions for NiTi alloys create an obstacle for wide application in biomedical industries. However, as PMMA polymer is biocompatible with lower density, this allows for easy processing steps, lower cost, and good formability [21,22,23]. Nevertheless, due to the polymer behavior of PMMA have inferior mechanical properties relative to NiTi alloy and the use of PMMA by itself to engineering application is limited. To combine both behaviors of alloy and polymer in an integrated manner for the formation of a composite, we have attempted to optimize the fabrication of NiTi–PMMA composite by a spin coating method, and we have analyzed shape memory performance as the function of physical properties and the function of thermal cycles.

The spin coating method is commonly used as an easy, fast, and controllable method for the formation onto substrates of high-quality PMMA resistance [24,25,26]. The spin coating with follow-up curing condition controls the coating thickness with a surface morphology of PMMA film on NiTi substrate of shape memory alloy (SMA) and super elastic (SE). Simultaneously, the concentration of PMMA polymer and various processing condition of the spin coating method allows uniform deposition of polymer films on the NiTi surface. In addition to that, the adhesion contact surface between NiTi and PMMA needs to be the subject of investigation. In this work, we implemented lines on the NiTi surface created by solid-state laser based on our previous findings [27,28,29]. The PMMA film coating is applied by spin coating for the deposition of films of uniform thickness and surface roughness. Toluene is frequently used as a solvent to dissolve PMMA in fundamental spin coating studies.

One of the interesting features of the NiTi–PMMA composite is thermally induced shape change. The fully reversible shape change in neat NiTi sample upon thermal cycling is called TWSME [30,31]. This effect is not an intrinsic property of shape memory alloys (SMAs) such as NiTi but must be induced through various procedures. Most commonly, the TWSME in NiTi or other SMAs is obtained through thermomechanical training [32,33]. Some other non-conventional ways of obtaining the TWSME are explained. The TWSME was induced by hydrogenation applied at the inner side of bent NiTi ribbons [34]. Additionally, the TWSME was obtained in Ni-rich NiTi shape memory ribbons by the combination of the all-round treatment and the R-phase transformation [35,36,37,38]. The TWSME in Fe-28.8 at.% Pd melt-spun ribbons arise due to a compositional gradient across the ribbon from the wheel contact side to the free contact side [39]. The TWSME is usually related to internal stress developed during a TWSME training procedure. In the case of NiTi–polymer composites, internal stress created at the composite interface due to a difference in the coefficients of thermal expansion and/or constrained shape recovery of NiTi is behind the reversible thermally induced shape change [40,41].

In this article, we investigate processing parameters for optimized coating deposition of PMMA film on NiTi surface with subsequent physical properties of the composite. The quality of PMMA film deposition and interface adhesion leads to the overall behavior of composite as the function of thermal cycles is examined. The shape memory effect of a composite in heating and cooling cycles with the radius of curvature is investigated by experimental method.

## 2. Materials and Methods

### 2.1. Fabrication of Composite System by Spin Coating Method

**Figure 2:**
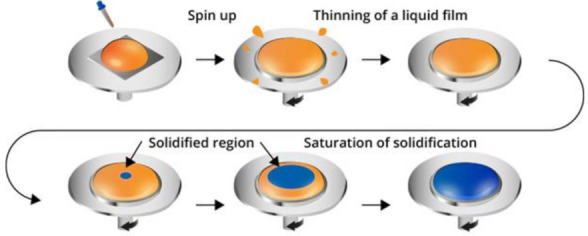
Spin coating deposition of PMMA on NiTi strip.

PMMA polymer powder is diluted with toluene in two different ratios 1:12 and 1:24 g/mL of concentration for spin coating deposition on NiTi surface. NiTi sample is designed in rectangular shape of 30 mm × 3 mm × 0.1 mm size, with follow-up laser engravings with a spacing of 100 μm in longitudinal direction [42,43]. The surface of the NiTi sample was patterned by laser engraving using marking laser ROD 20, NNM 18020V01, NARRAN, s.r.o. The type of laser used is a pulsed fiber ytterbium laser with diode-pumped machine. The effect of laser fluency energy was 20% of the maximum power 4 W, with a frequency of 20 kHz and a laser pulse length of 100 ns along the length of the NiTi. The laser engraving on NiTi surface causes the PMMA film to adhere on the NiTi surface by an interlocking mechanism. The spin coating was performed by the Laurell WS-400-6NPP spin coater with 650 series controller program software. The spin coating process is represented in a schematic diagram in Figure 2. The polymer solution of PMMA with toluene dissolved in two ratios, 1:12 and 1:24 g/mL, and was left for 12 h with a magnetic stirrer for uniform mixing. The concentration is added dropwise for the spin coating method for deposition of the film according to optimized parameters. The composite is post-baked at 80 °C, for 12 h, on a hot plate inside the furnace to release internal stresses and improve bonding between NiTi surface and PMMA film. Spin coating and post-processing conditions are determined parameters for obtaining PMMA films whose surface morphology either appears to be featureless or dominated by pinholes and defects [44,45,46]. On altering the concentration of PMMA powder with toluene solution, a thin film of PMMA is obtained on the NiTi surface. Table 1 represents the optimized parameters for uniform coating on the NiTi surface. Both shape memory alloy (SMA) and superelastic (SE) sheets are chosen for NiTi sample. The SMA–NiTi sample exhibits martensite phase at room temperature and is able to hold deformed shape with applied external force. However, SE–NiTi exhibits austenite phase at room temperature and springs back upon release of external force. Samples were obtained from the supplier SAES Getters group, Germany. The desired specimen was cut from the bulk commercial sample (78 mm × 360 mm) by laser cutting.

Both composites are cured at 80 °C to maintain homogeneity and uniformity of polymer layers and to release the residual stress.

### 2.2. Characterization of NiTi and Composite System Using Various Techniques

Surface features of NiTi and composites were examined by a Zeiss Imager Z1 Optical Microscope (OM, Zeiss, Oberkochen, Germany). Differential scanning calorimetry (DSC) analysis was carried out by DSC 25 (TA Instruments Trios v5.1.1.46572, New Castle, DE, USA) for the determination of the phase transition and glass transition temperature. The sample was heated at 10 °C/min from room temperature up to 150 °C and then maintained for one minute at this temperature, followed by cooling down at same rate to −100 °C. The thermo-mechanical response of the composite was investigated by thermo-mechanical analysis (TMA) using a LINSEIS L75 Cryo (Linseis, Sel, Germany) to observe the deflection in terms of displacement during the cooling and heating cycles. The displacement of the NiTi sample and its corresponding composite, on one and both sides, were considered at fixed load of 100 and 200 mN. The temperature was chosen in the range of −100 °C to +150 °C. The samples are cooled initially up to −100 °C with follow up heating up to 150 °C to observe the transformation peaks.

## 3. Results

### 3.1. Surface Morphology of PMMA Films Spin Coated on NiTi Substrates

**Figure 3:**
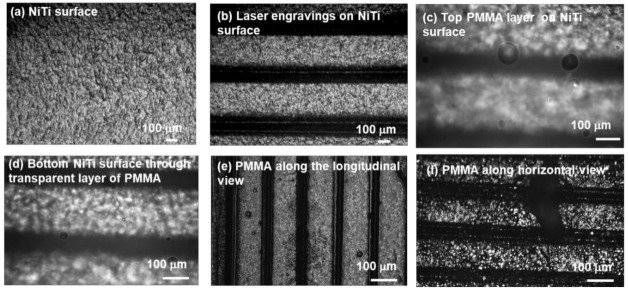
Image of PMMA coating on the NiTi substrate. (**a**) NiTi surface texture; (**b**) NiTi surface with laser line; (**c**) polymer-coated surface (transparent PMMA layer on the surface back surface); (**d**) front surface; (**e**) visual observation in longitudinal lines; (**f**) deposition of PMMA in horizontal lines.

**Figure 4:**
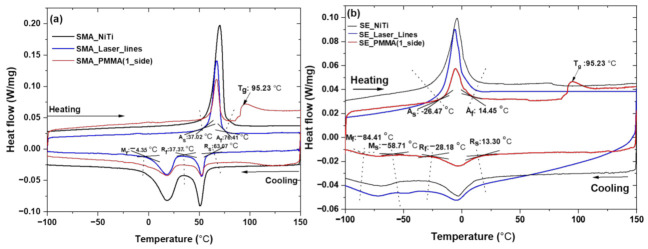
DSC curves (**a**) SMA, SMA_Laser_lines, SMA_PMMA (1_side), SE, and (**b**) SE_Laser_lines for SE_ PMMA (1_side) composites.

The surface of NiTi sample is rough and inhomogeneous, as shown in Figure 3a; however, the coating on this surface does not remain in an adhering state as a function of time. After curing, the PMMA film easily peels off from the NiTi surface. However, engraving lines by laser allows better adhesion of the PMMA film on the NiTi surface. The surface of the NiTi with lines is shown in Figure 4b. The NiTi surface can be seen through the transparent PMMA layer (Figure 3c,d). Composites were fabricated at a speed of 500 rpm for 30 s, at 8.3 mol %, showing better results than at 3200 rpm. However, on decreasing the concentration of a solution from 8.3 to 4.2 mol %, the quality of coating improves and is more tunable concerning control pinholes and defects. The ratio of the thickness between substrate NiTi to the deposition of PMMA film is 1:3. The post-baking process is more effective in the SMA-based composite compared to the SE composite, which releases internal deformation in SMA compared to SE.

### 3.2. Thermal Response of Individual Components of the Composite

**Figure 5:**
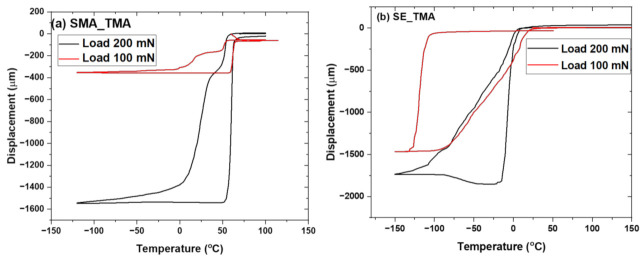
(**a**) SMA; (**b**) SE–NiTi shows thermo-mechanical analysis at two different loads of 100 and 200 mN.

The transformation temperatures of SMA and its corresponding composites, SE–NiTi samples, and its corresponding composites samples are shown in Figure 4a,b. Both SMA and SE samples are received (SE_NiTi, SMA_NiTi), the samples with laser engravings (SE_Laser_lines, SMA_Laser_lines) and corresponding PMMA film (SE_PMMA(1_side), SMA_PMMA(1_side)) coating on one side transform the temperature range from −100 to 150 °C. On cooling, samples show the presence of R phase and martensite phases for both SMA and SE samples. On heating, the austenite phase forms with a return to the original phase. The SMA_NiTi sample shows a martensite finish temperature (M_f_) of −4.35 °C, which overlaps with sample laser engravings (SMA_laser_lines) and with the PMMA coating on one side (SMA_PMMA(1_side)). The austenite finish temperature (A_f_) falls within 76.41 °C for sample and composite of PMMA with glass transition temperature (T_g_) of 95.23 °C. There is an overlap in the transformation temperature of the SE sample and its laser-annealed SE sample and corresponding composites. On cooling phase, start the R-phase transformation from 13.30 °C in the as-received SE sample to the composite, and simultaneously, on heating, the austenite finish temperature (A_f_) is 14.45 °C in the composite. The transformation temperature is summarized in Table 2. The NiTi samples with two static loads were investigated via thermo-mechanical analysis as the function of temperature. Figure 5a,b display the change in displacement for SE and SMA samples at two different loads of 100 and 200 mN. The sample shows change in displacement in bending position with fixed load of 100 and 200 mN at cooling and heating temperature. The samples return to original position after heating above the phase transformation temperature and above austenite finish temperature [27]. The shape recovery during heating shows the hysteresis behavior of the sample. SMA sample shows significant change in the displacement and hysteresis curve from −400 to −1600 μm. However, the SE–NiTi samples show less deformation with changing in applied load from 100 to 200 mN. The change in displacement varies only 200 μm.

### 3.3. Thermal Response of Composite as a Function of Heating and Cooling Cycle

**Figure 6:**
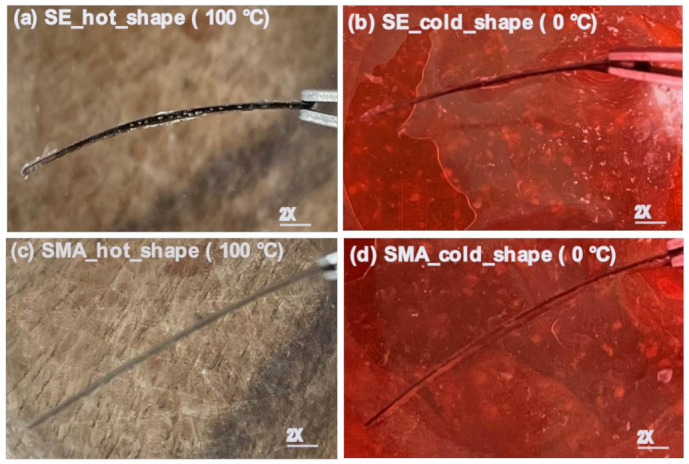
Shape recovery SE and SMA–PMMA composite (**a**–**d**) as the function of thermal cycles (heat, 100 °C and cold, 0 °C shape).

**Figure 7:**
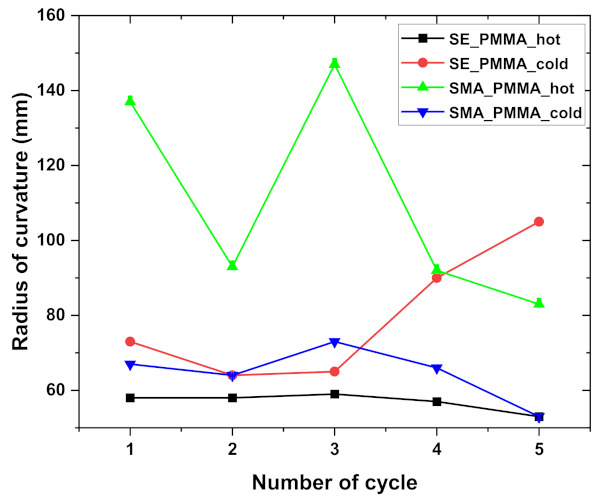
Radius of curvature as the function of the number of cycles for SE and SMA–PMMA composite.

The thermal response of composite of SMA–PMMA and SE–PMMA systems was investigated using heating-cooling cycles [47,48]. SE–PMMA composite shows a curved shape in hot cycles; however, the shape returns to a flat shape in cold cycles. However, the response of the SMA–PMMA composite shows completely reverse behavior compared to SE–PMMA composite. SMA–PMMA composite shows flat behavior at hot cycles (shape at 100 °C) and bent behavior in cold cycles (shape at 0 °C). SMA and SE–NiTi show no deformation because of the uniform coefficient of thermal expansion across the volume in the homogenous sample without any external load. The composite with polymer-coated NiTi shows the thermal response of the bending movement as the function of temperature in hot and cold cycles in Figure 6a–d. The radius of curvature from these cycles is calculated and plotted as the function of the number of cycles. It has been observed that with the number of cycles, there is a high fluctuation of the radius of curvature in the SMA–PMMA composite [49,50]. The change in curvature of the radius is calculated and plotted in Figure 7.

The dependency of the interlayer stress on the variable properties of PMMA–NiTi sample can be estimated by the following expression [51]

(1) $$\sigma =\frac{\left[\left(1-\nu_{2}\right)+\left(1-\nu_{1}\right)\xi \eta^{3}\right]E_{1}T_{1}}{3\left(1-\nu_{1}\right)\left(1-\nu_{2}\right)\left(1+\eta \right)\eta L_{s}^{2}}z$$

where *z* is the sample deflection, *L_s_* is the length of the sample, *E* is the Young’s modulus, *T* is the thickness, *η* = *T*_2_/*T*_1_ is the thickness ratio, *ξ* = *E*_2_/*E*_1_ is the modulus ratio, *ν* is the Poisson’s ratio, and subscripts 1 and 2 stand for NiTi and PMMA, respectively. Based on Equation (1), the interlayer stress and the Young’s modulus of the deposited layer can be easily determined [52]. Table 3 represents the interlayer stress values of composites at various cycles that derived from Figure 7.

### 3.4. Thermomechanical Behavior of Composite with Multiple Cycles

**Figure 8:**
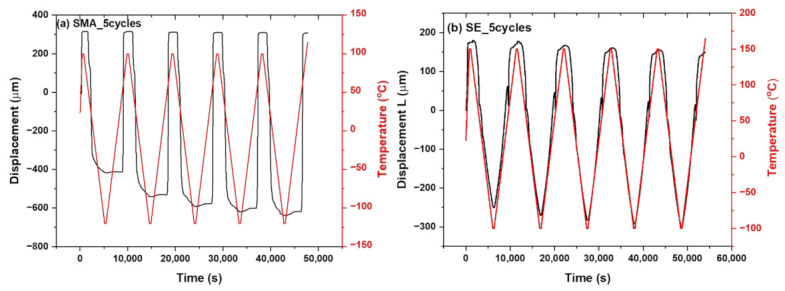
(**a**,**b**) Displacement and temperature profile for SMA and SE (5_cycles) as a function of time.

Thermomechanical behavior of SMA and SE–NiTi undergoes a three-point bending test by using a thermomechanical analyzer with a static load [53,54,55]. The investigation was performed on a static position with a constant load of 100 MN. The thermal cycles were varied for the SMA–NiTi in the range of −50 to 150 °C (Figure 8a), whereas temperature was varied for the SE–NiTi in the range of −100 to 100 °C (Figure 8b). To refine the response, the cycles were performed twice for each NiTi sample. Figure 8a,b represent the thermomechanical response of NiTi samples for both SMA and SE and their displacement (Delta L, μm) as the function of time and temperature.

**Figure 9:**
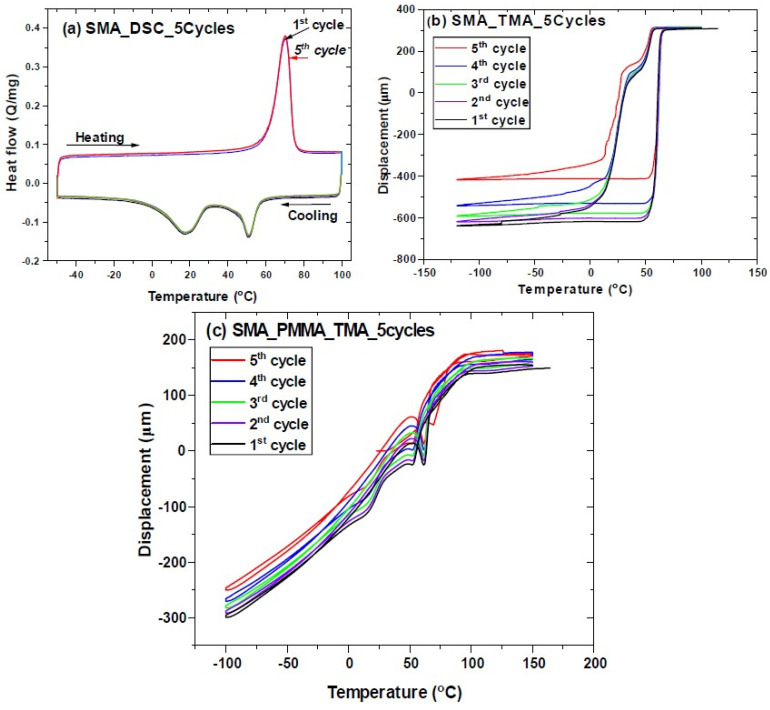
SMA multiple cycles (5th) for (**a**) DSC for SMA–NiTi, (**b**) TMA for SMA–NiTi, and (**c**) TMA for SMA–PMMA composite displacement as function of temperature.

**Figure 10:**
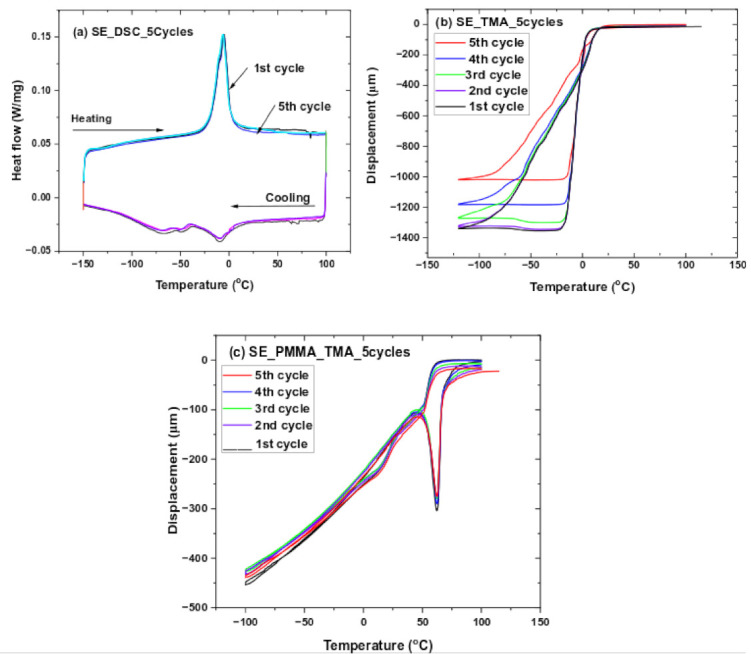
SE multiple cycles (5th) for (**a**) DSC for SE–NiTi, (**b**) TMA for SE–NiTi, and (**c**) TMA for SE–PMMA composite displacement as a function of temperature.

The multiple responses of the NiTi sample and composite system are performed in thermal and thermo-mechanical performance. SMA–NiTi underwent five thermal cycles to observe any shift in transformation temperatures. It has been observed that there is no shift in transformation temperatures on repeat of five cycles (Figure 9a). However, the thermo-mechanical response of SMA–NiTi samples shows there is a significant change in displacement of Delta L = −200 μm from 1st cycle to 5th cycle has been observed. Simultaneously SMA–PMMA composite samples show a change in displacement as the function of thermal cycles (Figure 10b). The sample returns to its original position during the heating cycle. There is a slight change in Delta L = −50 μm from 1st cycle to 5th cycle. There is a reduction in the change in behavior of the SMA–PMMA composite observed compared to SMA–NiTi sample. It may result due to bilayer of SMA and polymer PMMA in the composite system. The difference in CTE between SMA–NiTi and polymer is used as a lagging force for the deformation during thermal cycles (Figure 9C).

Simultaneously multiple responses for five cycles in the SE–NiTi samples and SE–PMMA composites were investigated by the thermal, thermo-mechanical response as the function of thermal cycles. Thermal cycles for SE–NiTi samples show no change in transformation temperatures during five cycles (Figure 10a). There is a strong overlap in the response of thermal peaks. The thermomechanical response of the SE–NiTi sample at 100 mN load shows the change in displacement. The displacement change varies as the function of multiple cycles is significant between the NiTi surface and PMMA in the SE–NiTi sample (Figure 10b). The change in displacement from 1st cycle to 5th cycle varies in the range of ΔL ≅ −300 µm. This may result in the accumulation of plastic strain during multiple cycles. However, the samples return to their original position without a shift in heating cycles. However, the SE–PMMA composite shows a slight change in displacement behavior during the thermomechanical cycles. There is a shift in heating cycles, there is remaining residual stress in the composite during multiple cycles, and it does not return to its original position. This response is observed in Figure 10c in the SE–PMMA composite system as the function of thermal cycles.

The DSC response of the SMA–NiTi sample and corresponding SMA samples with laser annealing and SMA–PMMA composites. However, the SE–NiTi sample and the sample with laser line and composites have a drastic change in transformation temperatures with a shift of more than ±50 °C.

Simultaneously NiTi–PMMA also has lower enthalpies of the A→R, R→M and M→A transformations. However, SE–PMMA composite shows similar in enthalpy in the SMA–PMMA composite [56,57]. The bent shape change and radius of curvature for SE and SMA composites are significant, with zero change (flat position) corresponding to the infinite position of the sample. An increase in radius of curvature of SMA–PMMA composite is observed as the function of the number of cycles (see Figure 7). Thermomechanical cycles with a static load condition of the NiTi sample show it recovered back to its original state without any change in displacement during heating. However, there is a slight shift in the original position in the case of composites that may be due to residual stress at the interface between the polymer and NiTi [58,59,60]. The interfacial stresses calculated for the composite at various cycles show strong fluctuation in values of stress for the PMMA–SMA composite at various cycles that correspond to the behavior of the shape change in Figure 8. However, the composite of PMMA–SE shows significant change in internal stress from the cooling to heating cycle that is well interpreted from the numerical findings from Table 3.

## 4. Conclusions

NiTi–PMMA composite samples could be able to change in shape with a temperature change. We have observed and measured that the curvature of the composite changes as a function of temperature. The experimental findings prove the dependency of the shape change and radius of the curvature to the temperature of thermal cycles. This may happen due to the accumulation of residual stress at the interface between the NiTi and polymer systems in the composite. The residual stresses arise due to differences in the combined parameters of Young’s modulus and CTE between NiTi and PMMA in the composite. The thermomechanical response of NiTi samples, both SE and SMA, shows recovery to the original position during thermal cycles at a constant load. However, the composites show a shift in position that may develop due to the accumulation of residual stress with the number of cycles.

## Figures and Tables

**Table 1:** Parameters for uniform coating of PMMA on NiTi surface by spin coating method and corresponding observation.

PMMA: Toluene (g/mL)	Spin Coating Parameters (Speed, Time of Duration)	Sample Thickness (mm)	Observation
1:12 (SE)	Speed: 3200 rpm, t: 30 s	0.216 ± 0.034	Samples with pinholes
	Speed: 500 rpm, t: 30 s	0.376 ± 0.052	Sample thickness improves
1:24 (SMA)	Speed: 500 rpm, t: 30 s	0.240 ± 0.023	Sample with fewer pinholes
	Speed: 100 rpm, t: 40 s Speed: 500 rpm t: 5 s	0.232 ± 0.030	fewer pinholes
1:24 (SE)	Step 1 + 2: Speed: 200 rpm, t: 40 s + 500 rpm, t: 20 s	0.492 ± 0.048	Thickness improves with better surface quality

**Table 2:** Transformation temperatures and enthalpies of NiTi and composite samples. (As such, the temperature accuracy of the Discovery DSC is ±0.025 °C, equivalent to the repeatability).

Sample	R_s_ (°C)	R_f_ (°C)	M_s_ (°C)	M_f_ (°C)	A_s_ (°C)	A_f_ (°C)	Enthalpy A→R (J/g)	Enthalpy R→M (J/g)	Enthalpy M→A (J/g)
SE–NiTi	13.30	−28.18	−58.71	−84.41	−26.47	14.45	2.7	2.0	10.7
SE–Laser-lines	13.30	−28.18	−58.71	−84.41	−26.47	14.45	2.7	2.0	10.7
SE–PMMA	13.30	−28.18	−58.71	−84.41	−26.47	14.45	2.7	2.0	10.7
SMA–NiTi	63.07	37.37	37.37	−4.35	37.02	76.41	4.7	6.6	18.0
SMA–Laser-lines	63.07	37.37	37.37	−4.35	37.02	76.41	4.7	6.6	18.0
SMA–PMMA	63.07	37.37	37.37	−4.35	37.02	76.41	4.7	6.6	18.0

The R-phase is found in nitinol, a shape memory alloy. It is a martensitic phase in nature, but it is not the martensite that is responsible for the shape memory and superelastic effect. R_s_: R-phase start temperature; R_f_: R-phase finish temperature; M_s_: martensite phase start temperature; M_f_: martensite phase finish temperature; A_s_: Austenite phase start temperature; A_f_: austenite phase finish temperature.

**Table 3:** Interlayer stress for various composites with PMMA at various cycles.

Sample Case	Cycle Number				
	Cycle 1 (Stress in MPa)	Cycle 2 (Stress in MPa)	Cycle 3 (Stress in MPa)	Cycle 4 (Stress in MPa)	Cycle 5 (Stress in MPa)
SMA_PMMA (100 °C)	0.54 ± 0.001	0.81 ± 0.002	0.52 ± 0.001	0.81 ± 0.002	0.84 ± 0.003
SMA_PMMA (0 °C)	0.71 ± 0.004	0.73 ± 0.004	0.62 ± 0.003	0.73 ± 0.004	0.8 ± 0.01
SE_PMMA (100 °C)	1.26 ± 0.008	1.24 ± 0.008	1.23 ± 0.008	1.27 ± 0.008	1.29 ± 0.008
SE_PMMA (0 °C)	0.61 ± 0.003	0.73 ± 0.004	0.72 ± 0.004	0.53 ± 0.002	0.44 ± 0.002

## Data Availability

Data will be available on the request of readers on writing to authors.

## References

[1] Huang W.M., Ding Z., Wang C.C., Wei J., Zhao Y., Purnawali H. (2010). Shape memory materials. Mater. Today.

[2] Wei Z.G., Sandstroröm R., Miyazaki S. (1998). Shape-memory materials and hybrid composites for smart systems—Part I shape-memory materials. J. Mater. Sci..

[3] Otsuka K., Wayman C.M. (1998). Shape Memory Materials.

[4] Sun L., Huang W., Ding Z., Zhao Y., Wang C., Purnawali H., Tang C. (2012). Stimulus-responsive shape memory materials: A review. Mater. Des..

[5] Jani J.M., Leary M., Subic A., Gibson M.A. (2014). A review of shape memory alloy research, applications and opportunities. Mater. Des..

[6] Yamauchi K., Ohkata I., Tsuchiya K., Miyazaki S. (2011). Shape Memory and Superelastic Alloys: Applications and Technologies.

[7] Morgan N.B. (2004). Medical shape memory alloy applications—The market and its products. Mater. Sci. Eng. A.

[8] Fang C., Wang W. (2020). Shape Memory Alloys for Seismic Resilience.

[9] Hassani F.A., Shi Q., Wen F., He T., Haroun A., Yang Y., Feng Y., Lee C. (2020). Smart materials for smart healthcare-moving from sensors and actuators to self-sustained nanoenergy nanosystems. Smart Mater. Med..

[10] Gök M.O., Bilir M.Z., Gürcüm B.H. (2015). Shape-memory applications in textile design. Procedia–Soc. Behav. Sci..

[11] Pineda-Castillo S.A., Stiles A.M., Bohnstedt B.N., Lee H., Liu Y., Lee C.-H. (2022). Shape Memory Polymer-Based Endovascular Devices: Design Criteria and Future Perspective. Polymers.

[12] Song J.J., Chang H., Naguib H.E. (2015). Biocompatible shape memory polymer actuators with high force capabilities. Eur. Polym. J..

[13] Huang W.M., Zhao Y., Wang C.C., Ding Z., Purnawali H., Tang C., Zhang J.L. (2012). Thermo/chemo-responsive shape memory effect in polymers: A sketch of working mechanisms, fundamentals and optimization. J. Polym. Res..

[14] van der Schaar P.J., Dijksman J.F., Gast H.B.-D., Shimizu J., van Lelyveld N., Zou H., Iordanov V., Wanke C., Siersema P.D. (2013). A novel ingestible electronic drug delivery and monitoring device. Gastrointest. Endosc..

[15] Khodagholy D., Gelinas J.N., Thesen T., Doyle W.K., Devinsky O., Malliaras G., Buzsáki G. (2015). NeuroGrid: Recording action potentials from the surface of the brain. Nat. Neurosci..

[16] Kim D.-H., Lu N., Ma R., Kim Y.-S., Kim R.-H., Wang S., Wu J., Won S.M., Tao H., Islam A. (2011). Epidermal electronics. Science.

[17] Lee W.W., Tan Y.J., Yao H., Li S., See H.H., Hon M., Ng K.A., Xiong B., Ho J.S., Tee B.C.K. (2019). A neuro-inspired artificial peripheral nervous system for scalable electronic skins. Sci. Robot..

[18] Samal S., Prado Ede Tyc O., Sittner P. (2018). Shape setting in super-elastic NiTi ribbon. IOP Conf. Ser.: Mater. Sci. Eng..

[19] Xiang Z., Wang H., Pastorin G., Lee C. (2015). Development of a flexible and disposable microneedle-fluidic-system with finger-driven drug loading and delivery functions for inflammation treatment. J. Microelectromechan. Syst..

[20] Vokoun D., Sysel P., Heller L., Kadeřávek L., Svatuška M., Goryczka T., Kafka V., Šittner P. (2016). NiTi-Polyimide Composites prepared using Thermal Imidization Process. JMEPEG.

[21] Zhang W., Lin S., Wang C., Hu J., Li C., Zhuang Z., Zhou Y., Mathies R.A., Yang C.J. (2009). PMMA/PDMS valves and pumps for disposable microfluidics. Lab Chip.

[22] Fung C.K., Zhang M.Q., Chan R.H., Li W.J. A PMMA-based micro pressure sensor chip using carbon nanotubes as sensing elements. Proceedings of the 18th IEEE International Conference on Micro Electro Mechanical Systems (MEMS).

[23] Shiraishi N., Kimura M., Ando Y. (2014). Development of PMMA-based gas sensor and its evaluation using a VOC dilution flow system. Microelectron. Eng..

[24] Çapan I., Tanmcr C., Erdoğan M., Hassan A.K. (2009). Characterisation and vapour sensing properties of spin coated thin films of anthracene labelled PMMA polymer. Mater. Sci. Eng. C.

[25] Çapan İ., Tarımcı Ç., Hassan A.K., Tanrısever T. (2009). Characterisation and optical vapour properties of PMMA thin films. Mater. Sci. Eng. C.

[26] Stefanescu E.A., Tan X., Lin Z., Bowler N., Kessler M.R. (2011). Multifunctional fiberglass-reinforced PMMA-BaTiO_3_ structural/dielectric Composites. Polymer.

[27] Humbeeck Van J., Stalmans R., Otsuka K., Wayman C.M. (1998). Thermomechanical Properties of SMA: Shape Memory Materials.

[28] Zhang X.M., Fernandez J., Guilemany J.M. (2006). Role of external applied stress on the two-way shape memory effect. Mater. Sci. Eng. A.

[29] Leu C.C., Vokoun D., Hu C.T. (2002). Two-way shape memory effect of TiNi alloys induced by hydrogenation. Metall. Mater. Trans. A.

[30] Wu Z.H., Vokoun D., Leu C.C., Hu C.T. (2017). A two-way shape memory study on Ni-rich NiTi shape memory alloy by combination of the all-round treatment and the R-phase transformation. J. Mater. Eng. Perform..

[31] Šittner P., Landa M., Lukáš P., Novák V. (2006). R-phase transformation phenomena in thermomechanically loaded NiTi polycrystals. Mech. Mater..

[32] Chang C.Y., Vokoun D., Hu C.T. (2001). Two-Way Shape Memory Effect of NiTi Alloy Induced by Constraint Aging Treatment at Room Temperature. Metall. Mater. Trans. A.

[33] Vokoun D., Hu C.T. (2002). Two-way shape memory effect in Fe-28.8 at.% Pd melt-spun ribbons. Scr. Mater..

[34] Samal S., Blanco I. (2021). Investigation of Dispersion, Interfacial Adhesion of Isotropic and Anisotropic Filler in Polymer Composite. Appl. Sci..

[35] Samal S., Molnárová O., Průša F., Kopeček J., Heller L., Šittner P., Škodová M., Abate L., Blanco I. (2021). Net-Shape NiTi Shape Memory Alloy by Spark Plasma Sintering Method. Appl. Sci..

[36] Samal S., Tyc O., Cizek J., Klecka J., Lukáč F., Molnárová O., de Prado E., Weiss Z., Kopeček J., Heller L. (2021). Fabrication of Thermal Plasma Sprayed NiTi Coatings Possessing Functional Properties. Coatings.

[37] Semaltianos N.G. (2007). Spin-Coated PMMA films. Microelectron. J..

[38] Naghashian S., Fox B.L., Barnett M.R. (2014). Actuation curvature limits for a composite beam with embedded shape memory alloy wires. Smart Mater. Struct..

[39] Dahnke C., Reeb A., Pottmeyer F., Weidenmann K.A., Tekkaya A.E. (2019). Thermomechanical behavior of shape memory alloy metal matrix composite actuator manufactured by composite extrusion. Smart Mater. Struct..

[40] Lester B.T., Baxevanis T., Chemisky Y., Lagoudas D.C. (2015). Review and Perspectives: Shape Memory Alloy Composite Systems. Acta Mech..

[41] Samal S., Tyc O., Heller L., Šittner P., Malik M., Poddar P., Catauro M., Blanco I. (2020). Study of Interfacial Adhesion between Nickel-Titanium Shape Memory Alloy and a Polymer Matrix by Laser Surface Pattern. Appl. Sci..

[42] Stachiv I., Alarcon E., Lamac M. (2021). Shape Memory Alloys and Polymers for MEMS/NEMS Applications: Review on Recent Findings and Challenges in Design, Preparation, and Characterization. Metals.

[43] Clyne T.W. (1996). Residual Stresses in Surface Coatings and Their Effects on Interfacial Debonding. Key Eng. Mater..

[44] Pryor R.W. (2011). Multiphysics Modeling Using Comsol: A First Principles Approach.

[45] Samal S. (2022). Interface failure and delamination resistance of fiber-reinforced geopolymer composite by simulation and experimental method. Cem. Concr. Compos..

[46] Cohades A., Michaud V. (2018). Shape memory alloy in fiber-reinforced polymer composites. Adv. Ind. Eng. Polym. Res..

[47] Park J., Headings L.M., Dapino M.J., Baur J.W., Tandon G.P. (2015). Investigation of interfacial shear stresses, shape fixity, and actuation strain in composites incorporating shape memory polymers and shape memory alloys. Front. Mater..

[48] Wilson S.A., Jourdain R.P.J., Zhang Q., Dorey R.A., Bowen C.R., Willander M., Ul Wahab Q., Willander M., Al-hilli S.M., Nur O. (2007). New materials for micro-scale sensors and actuators: An engineering review. Mater. Sci. Eng. R Rep..

[49] Kohl M., Dittmann D., Quandt E., Winzek B. (2000). Thin film shape memory microvalves with adjustable operation temperature. Sens. Actuators.

[50] Sun Z., Xu Y., Wang W. (2019). Experimentation of the Bilinear Elastic Behavior of Plain-Woven GFRP Composite with Embedded SMA Wires. Polymers.

[51] Tsoi K.A., Schrooten J., Zheng Y., Stalmans R. (2004). Part II. Thermomechanical characteristics of shape memory alloy composites. Mater. Sci. Eng..

[52] Parthenios J., Psarras G., Galiotis C. (2001). Adaptive composites incorporating shape memory alloy wires. Part 2: Development of internal recovery stresses as a function of activation temperature. Compos. Part A Appl. Sci. Manuf..

[53] Bollas D., Pappas P., Parthenios J., Galiotis C. (2007). Stress generation by shape memory alloy wires embedded in polymer composites. Acta Mater..

[54] Michaud V. (2004). Can shape memory alloy composites be smart?. Scr. Mater..

[55] Stachiv I., Sittner P. (2018). Nanocantilevers with Adjustable Static Deflection and Significantly Tunable Spectrum Resonant Frequencies for Applications in Nanomechanical Mass Sensors. Nanomaterials.

[56] Stachiv I., Gan L. (2019). Simple Non-Destructive Method of Ultrathin Film Material Properties and Generated Internal Stress Determination Using Microcantilevers Immersed in Air. Coatings.

[57] Taheri-Behrooz F., Taheri F., Hosseinzadeh R. (2011). Characterization of a shape memory alloy hybrid composite plate subject to static loading. Mater. Des..

[58] Lei H., Wang Z., Zhou B., Tong L., Wang X. (2012). Simulation and analysis of shape memory alloy fiber reinforced composite based on cohesive zone model. Mater. Des..

[59] Tsoi K.A., Stalmans R., Schrooten J. (2002). Transformational Behavior of Constrained Shape Memory Alloys. Acta Mater..

[60] Taya M., Liang Y., Namli O.C., Tamagawa H., Howie T. (2013). Design of two-way reversible bending actuator based on a shape memory alloy/shape memory, polymer composite. Smart Mater. Struct..

